# Hospitalization and Housing: A Qualitative Study Exploring the Perspectives of Hospitalized Patients Experiencing Housing Insecurity

**DOI:** 10.7759/cureus.46367

**Published:** 2023-10-02

**Authors:** Erin L Bredenberg, Julie Knoeckel, Kathryn Havranek, Lauren McBeth, Sarah Stella, Mackenzie Garcia, Ellen Sarcone, Greg Misky

**Affiliations:** 1 Hospital Medicine, University of Colorado School of Medicine, Aurora, USA; 2 Hospital Medicine, Denver Health and Hospitals, Denver, USA; 3 Internal Medicine, New York University (NYU) Langone Health, New York, USA; 4 Internal Medicine, Emory University School of Medicine, Atlanta, USA; 5 Hospital Medicine, University of Colorado School of Medicine, Denver, USA

**Keywords:** qualitative research, hospitalization, social determinants of health (sdoh), patients experiencing homelessness, housing

## Abstract

Although housing insecurity has clear negative impacts on health, little is known about how it impacts patients’ experience of hospitalization. In this qualitative study, we interviewed 22 hospitalized patients experiencing housing insecurity. The following three major themes emerged: 1) adverse social and environmental factors directly contribute to hospitalization, 2) lack of tailored care during hospitalization leaves patients unprepared for discharge, and 3) patients have difficulty recuperating after a hospital stay, leading to the risk of rehospitalization. Within these themes, participants described the roles of extreme physical and psychological hardship, chaotic interpersonal relationships, substance use, and stigma affecting participants’ experiences before, during, and following hospitalization. Our results, based directly on the patient experience, suggest a need for hospital systems to invest in universal in-hospital screening for housing insecurity, incorporation of trauma-informed care, and robust partnerships with community organizations. Future research should explore the feasibility and impact of these interventions.

## Introduction

Homelessness is a major public health issue and has been identified as “the health equity challenge of our time” [1]. People experiencing homelessness (PEHs) experience inequities in health outcomes including higher mortality and higher rates of acute healthcare utilization as compared with their housed counterparts [2]. PEHs are hospitalized more often than those who are stably housed, and when hospitalized, they have longer lengths of stay, higher readmission rates (up to 14 percentage points higher than housed counterparts), and poorer quality in-hospital experiences [3-5]. Less is known about the outcomes of hospitalization for people experiencing housing insecurity without frank homelessness, due in part to the challenges defining and measuring “unstable” or “vulnerable” housing [6]; however, there is limited evidence for an association between negative health outcomes and insecure housing [7,8]. Although housing insecurity negatively impacts acute care-related health outcomes, it often is not routinely and adequately addressed during hospitalization, leading to inadequate resource connection and discharge planning for PEHs and those at risk of housing loss [9].

As the deleterious impacts of homelessness become more widely recognized, hospital systems increasingly accept the need to develop interventions to address the specific needs of PEHs [10,11]. Hospitalization represents a unique opportunity during which critical interventions to address social determinants of health can be implemented. To ensure that such interventions are welcome, needed, and efficacious, it is crucial to understand the perspectives of people experiencing housing insecurity during the time of hospitalization. While prior studies have addressed the perspectives of hospital-based providers caring for PEHs [12], the views of PEHs themselves during the time of hospitalization have not been explored. Furthermore, while the perspectives of PEHs have been described in other settings, the views of those not experiencing frank homelessness but living along a spectrum of housing-insecure situations have not specifically been elicited [13-15].

There is an urgent need to concretely address health inequities affecting those with housing insecurity around the time of hospitalization [16]. Understanding and incorporating the patient perspective is a critical step in the design and implementation of effective interventions, particularly when a stigmatized population is affected [17]. In this study, we describe the perspectives of individuals who were hospitalized while experiencing housing insecurity to inform future interventions to reduce health disparities in the housing-insecure community.

This work was presented at the 2020 Society of General Internal Medicine National Meeting in oral abstract form and the 2020 Public Health in the Rockies regional meeting in poster form in 2020.

## Materials and methods

Study design

This was a qualitative study of hospitalized patients experiencing housing insecurity. We conducted 22 in-depth, semi-structured key informant interviews with individual patients at two large hospitals. The interview guide was created by the study team, the majority of whom were clinicians or trainees with experience caring for patients experiencing homelessness in an acute care setting, with input from community members with expertise in the subject. We used the Consolidated Criteria for Reporting Qualitative Health Research (COREQ) as a framework to report findings for this study [18].

Setting

This study was conducted at a major academic center and an urban safety-net hospital in a major metropolitan area in the United States.

Data collection and participants

All patients aged 18 or older who were hospitalized between July 2019 and March 2020 at one of the two study sites were eligible for inclusion. We excluded non-English speaking and incarcerated individuals. This study was approved by the Colorado Multiple Institutional Review Board (COMIRB) under protocol #18-1158.

Patients were selected randomly to approach to screen for inclusion, and the housing status of patients approached for interview was unknown at the time of approach. A random number generator was used to select potential participants from a list of medical inpatients to approach for inclusion in the study. This approach was taken to ensure inclusion of patients experiencing a broad range of unstable housing situations and minimize interviewer bias about potential participants’ housing status. Participants were approached in their hospital room and, after consenting to participation, were screened for the presence of housing insecurity using a four-question screening survey (Appendix 1). Participants who were identified as housing insecure (i.e., homeless or vulnerably housed) based on screening survey results completed an audio-recorded semi-structured interview with qualitative and quantitative components. Patients who reported living on the streets, in a homeless shelter, in a vehicle, or in abandoned buildings were considered to be experiencing homelessness. Those who reported “couch-surfing,” being at risk of losing housing, or having moved four or more times in the past year were considered vulnerably housed. Interviews were performed by one of six trained interviewers (physicians, medical students, and nurse practitioner students) who had no prior relationship with the patients. Interviews took place between July 2019 and March 2020 in patient’s hospital rooms; recruitment was ongoing until a sufficient number of patients had been interviewed to achieve thematic saturation as defined by the point at which no new concepts emerged. The interview guide was comprised of a total of 27 questions, with 11 of these being open-ended (Appendix 2). The average interview length was 27 minutes. Participants were compensated with a $5 gift card.

Analysis

Recorded interviews were professionally transcribed verbatim and deidentified. The transcripts were inductively coded and analyzed by a four-person coding group that included interviewers and non-interviewers (E.B., L.M., J.K., K.H.). The team consisted of two hospital medicine physicians, an experienced research assistant with extensive qualitative experience, and a medical student. We used a phenomenological approach to analyze interview transcripts for thematic content [19]. A codebook was developed by having two members of the coding team (E.B., J.K.) independently review several transcripts to create an initial list of codes (i.e., descriptors that captured the concepts and ideas discussed in the interviews). The codebook was finalized through an iterative review of transcripts and discussion between all four coding team members. Coding was completed with any discrepancies resolved by consensus, with the use of ATLAS.ti (Version 8) software for data management. The coding team then analyzed coded data for emerging themes related to housing insecurity, health, and hospitalization. Interviews were conducted until thematic saturation was reached. Following the initial coding process, the two clinician members of the coding team (E.B., J.K.) performed a more focused analysis using a “Sort, Sift, and Think” approach in order to better address the original research question designed to explore patient perspectives specifically around the time of hospitalization [20]. In this approach, researchers “dive in” and then “step back” from the data to “arrive at an evidence-based meeting point that is a hybrid story of data content and researcher knowledge” [20]. This approach allowed for an effective re-analysis of the data without loss of the integrity of the patient voice or compromise of the analytic process.

## Results

**Figure 1:**
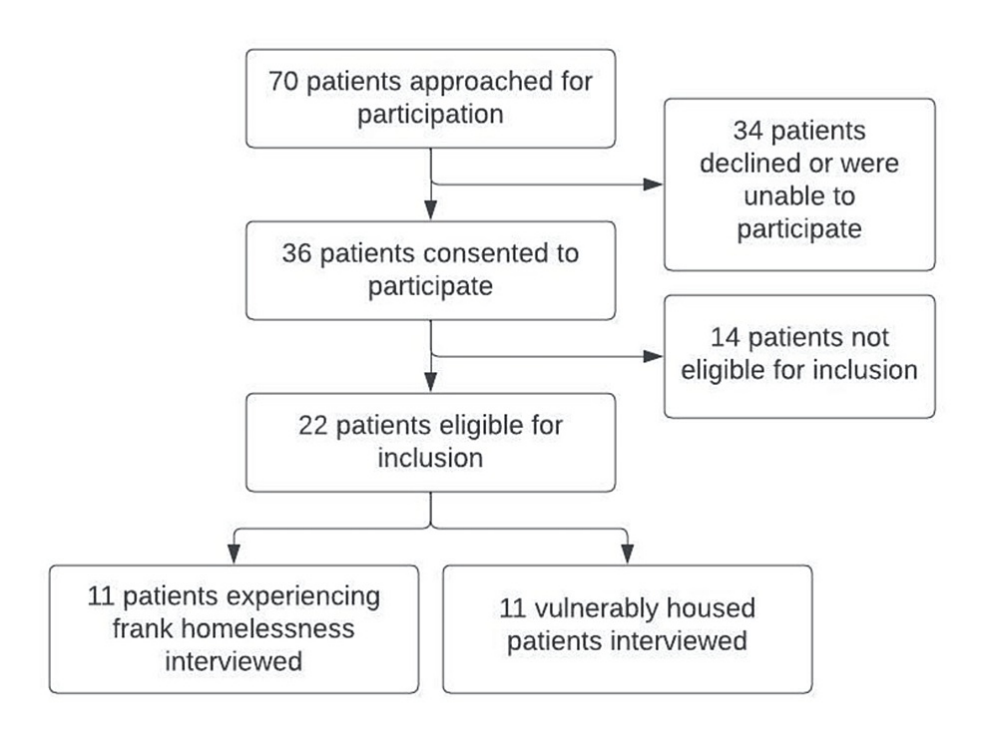
Recruitment flow sheet

A total of 70 patients were approached to participate, of whom 36 consented to participate. Of those who consented, 14 reported stable housing and therefore were not eligible for participation in the study. Twenty-two individuals who met criteria for housing insecurity were interviewed: 9 of those were hospitalized at the major academic hospital and 13 at the safety-net hospital. Eleven were vulnerably housed, and 11 were experiencing frank homelessness (Figure 1, Table 1). Most participants experiencing homelessness reported inadequate access to food, water, electricity, and toilet facilities, whereas a minority of vulnerably housed participants reported lack of access to these basic facilities (Table 2).

**Table 1:** Patient demographics (n=22)

Characteristics	N (%)
Sex	
Female	10 (45)
Male	12 (55)
Race	
White	15 (68)
Black	3 (14)
Multi-racial	1 (5)
Unknown	3 (14)
Ethnicity	
Hispanic/Latino	5 (23)
Non-Hispanic/Latino	17 (77)
Age (years)	
18-24	1 (5)
25-54	17 (77)
55-64	2 (9)
65+	2 (9)

**Table 2:** Expanded patient characteristics *One participant did not answer the question

	Frank Homelessness	Vulnerably Housed	Total
	N (%)	N (%)	N (%)
Believes housing affects health at least “somewhat”	11 (100)	10 (100)*	21 (100)*
Experienced inadequate heat in the last year	10 (91)	2 (18)	12 (55)
Experienced inadequate electricity in the last year	9 (82)	2 (18)	11 (50)
Experienced inadequate water in the last year	9 (82)	2 (18)	11 (50)
Experienced inadequate toilet access in the last year	11 (100)	2 (18)	13 (59)
Experienced inadequate food access in the last year	9 (82)	1 (9)	10 (45)
Reports at least one reliable personal relationship	6 (55)	9 (82)	15 (68)
At least “somewhat comfortable” discussing housing status with providers	6 (60)*	7 (64)	13 (62)*
Had medications lost or stolen in the last year	10 (91)	4 (36)	14 (64)
Offered resources during this hospitalization	7 (64)	5 (45)	12 (55)

We identified three main themes and four subthemes (Table 3).

**Table 3:** Themes and subthemes

Theme 1: Adverse social and environmental factors directly contribute to hospitalization
Subtheme 1a: The lived experience of housing insecurity is associated with extreme physical and psychological hardships
Subtheme 1b: There is a bidirectional relationship between substance use and housing insecurity that impacts patients’ need for hospitalization
Theme 2: Lack of tailored care during hospitalization leaves patients unprepared for discharge
Subtheme 2a: Fear of stigmatization by healthcare professionals interferes with patients’ ability to engage with the healthcare system
Subtheme 2b: Hospitalization may incite economic destabilization for those with vulnerable housing
Theme 3: Patients have difficulty recuperating after a hospital stay, leading to risk of entering a vicious cycle of rehospitalization

Theme 1: Adverse Social and Environmental Factors Directly Contribute to Hospitalization

While participants in this study reported a diversity of experiences, the deleterious impact of unstable housing on health was overwhelmingly supported. Many spoke about the importance of having their hospital medical providers understand the realities of their living situations.

Subtheme 1a: The lived experience of housing insecurity is associated with extreme physical and psychological hardships.

Participants experiencing homelessness spoke about extraordinary physical hardships they faced and how these experiences negatively impacted their health and well-being. Constant threats to safety including physical violence, environmental exposures, and victimization emerged as driving factors contributing to poor health.

It can be dangerous. People can hurt you and things like that. Some people are very mean and horrible out there.

Others described the many ways in which a homeless shelter environment could be detrimental to maintaining good health.

I really don't want to go back to the shelter. It's just that it's so noisy, and you get very little sleep there because a lot of people just go there to chitchat until midnight or 1:00 in the morning, then all the sudden at 4:00 in the morning they're waking you up.

Though immediate threats to physical well-being were much less frequently encountered by the vulnerably housed participants, many in this group emphasized psychological distress related to economic hardship, uncertainty, and reliance on sometimes volatile interpersonal relationships to maintain housing. Almost all participants relayed stories of chaotic interpersonal relationships as central in the generation of their housing insecurity. Many vulnerably housed participants reported their ability to maintain housing was contingent on the status of relationships with friends or family, often at the expense of mental health.

We went back to his mom's and we stayed there, but that relationship is non-existent, [and] it affected us because whenever she got mad she could just kick us out. [We] always had to worry ‘Where are we going to go if this happens?’ And it can cause stress on your body.

Subtheme 1b: There is a bidirectional relationship between substance use and housing insecurity which impacts patients’ need for hospitalization.

Substance use was a pervasive experience among participants, and some reported that their current hospitalization was a direct sequelae of substance use. Participants depicted a bidirectional impact of substance use and housing insecurity, with many describing substance use as an essential coping mechanism for dealing with the physical and psychological challenges of homelessness. A few went so far as to describe their substance use as inescapable as long as they continued to experience homelessness.

Everyone turns to drugs because of their situation because it gets so stressful not knowing if you got [a] place to sleep, if you're going to be safe, warm, or fed.

I know most people who wanted to be sober, that's their concern. They don't want to go back to where they were because they'll wind up using.

Notably, a few patients described the potential to break the cycle of homelessness and potentially avoid further hospitalization by being connected with sober living programs at discharge.

I'm going to be going to sober living, so that's better than being on the street, you know what I mean? And I want to be sober, and the hospital's totally helping me with that which is really great.

Theme 2: Lack of Tailored Care During Hospitalization Leaves Patients Unprepared for Discharge

Vulnerably housed and homeless participants alike described fears surrounding discharge. Many participants described concerns about effective recuperation post-hospitalization and being discharged before feeling ready to do so. Some described being unwilling to discuss housing status and how it impacted their ability to follow recommended care with providers due to concerns about stigma, though a few felt that discussing housing status during hospitalization opened the potential to address specific barriers after discharge.

Subtheme 2a: Fear of stigmatization by healthcare professionals interferes with patients’ ability to engage with the healthcare system.

Many participants cited frequent and distressing experiences with stigma related to appearing “homeless” and to substance use. Fear of further stigmatization was described as a powerful deterrent for engagement with the healthcare system. While specific experiences differed, the general experience of stigma, especially from healthcare professionals, was nearly ubiquitous.

It makes it hard sometimes, you don’t want to go into the hospital because you just feel like you're going to get treated like s***, so you just stay away for as long as you can.

Stigma was a major driver of challenges in accurately screening for housing insecurity in the hospital. Several participants expressed reluctance to discuss housing status with their inpatient providers due to a fear of judgment. For instance, one participant reported providing an inaccurate address at the time of registration to avoid being categorized as homeless. Another reckoned with the need to overcome a sense of shame in disclosing homelessness to gain access to resources. Many participants expressed significant gratitude for care when they perceived the absence of stigma.

I’m appreciative of the fact that whether I was homeless or not, it didn’t seem to affect their care for me or their attitude towards me.

Participants who felt comfortable discussing their housing status with medical providers endorsed that having their housing status more fully understood allowed for more personalized and achievable plans at the time of discharge. A few noted that they might prefer to discuss this sensitive issue only with a subset of providers whom they perceived as empathetic.

I’d be okay discussing it [housing status] with them if they were genuinely concerned. And trying to figure out some way to help me.

Subtheme 2b: Hospitalization may incite economic destabilization for those with vulnerable housing.

Among the complex, multifactorial reasons for entry into housing insecurity, interactions with the healthcare system were frequently identified as a contributor. For some vulnerably housed participants, the economic threat posed by recurrent or prolonged admissions was the primary driver of housing insecurity.

That's $1500 a month, that's the going rate plus the damage deposit and the first month’s rent. It gets to be just a little bit out of your reach. If I wasn’t sick here like this right now, I’d be trying to do day labor or something.

Likewise, the presence of new health conditions or disability imparted a destabilizing effect on employment and economic status, jeopardizing previously stable housing. Some participants endorsed that their hospital care team was not aware of the challenges they were facing after hospitalization and described immense stress about the competing priorities of managing new housing insecurity with concurrent health challenges. There was uncertainty about whether prioritizing their health by remaining in the hospital as recommended was truly in their best interest.

I had a doctor who just didn’t understand. They wanted to keep me in the hospital, and I kept explaining, ‘I've got a job. I've got to get back to my job.’ And they would not hear it.

Theme 3: Patients Have Difficulty Recuperating After a Hospital Stay, Leading to Risk of Entering a Vicious Cycle of Rehospitalization

Many participants experiencing homelessness described a number of perceived and experienced barriers to effective transitions of care, potentially leading to worsening health and poor outcomes such as hospital readmission.

I’m supposed to take diuretics all the time, so, I’m having to constantly pee. But I don’t have access to a toilet. That creates a real problem for me, so I have to choose to not take my diuretics. It’s causing me to bloat up, I retain all my water. I have to come back in here, like a never-ending cycle.

Other concerns ranged from inability to access a shelter bed due to late timing of discharges to inadequate access to reliable transportation for follow-up appointments. Furthermore, the experience of loss or theft of prescription medications was frequently reported among participants.

Many participants specifically described the impossibilities of effectively recuperating from hospitalization due to inadequate or unsafe living conditions. 

Sleeping in the car, I am too tall to be comfortable. I am moving all night, shifting, trying to get comfortable. I just had a right hip replacement, so that’s very difficult to do.

Some participants expressed concerns about physical and psychological safety specifically related to relationships with roommates or family at the time of discharge from the hospital.

I used to think that I could go to my daughter's, but I've come to realize that she's not comfortable seeing me in what she considers a weakened state. She doesn’t like to see my walker. She'll hide it from me, so I can't find it because she thinks that I need to be on my feet. There were three different times I fell because I didn’t have it.

## Discussion

**Figure 2:**
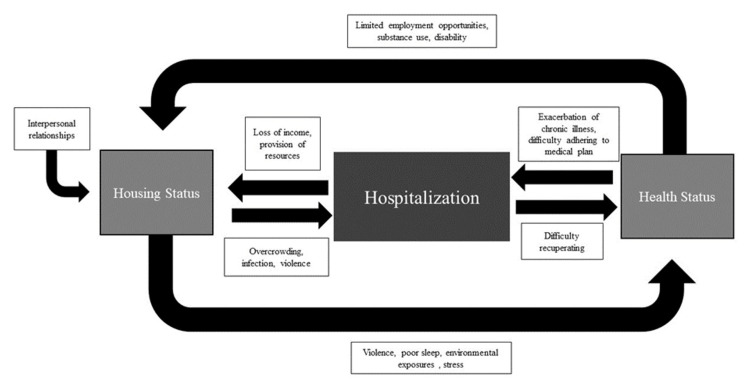
Interactions between housing, health, and hospitalization This image was created by the authors

For patients experiencing any degree of housing insecurity, the context surrounding an episode of hospitalization is critical for healthcare providers to consider. Patients experiencing a range of housing-insecure living situations are at risk of entering a vicious cycle of poor health, hospitalization, and ongoing housing insecurity, with substance use, economic instability, and chaotic interpersonal relationships as notable driving factors. The interactions between housing insecurity, health, and hospitalization are complex, and we have proposed a model of the interplay between these factors informed by our results in Figure 2. Our findings suggest recommendations to improve care of patients experiencing housing insecurity along three increasingly broad levels of care.

Recommendations for inpatient and transitional care

Patients in our study unequivocally expressed the negative impacts of housing insecurity on health, and many felt that their healthcare providers could provide better care when housing status was considered. Implementation of routine, standardized, patient-centered housing assessment for all hospitalized patients may help identify and direct patients to appropriate resources prior to discharge; one study of an emergency department-based program to screen for housing insecurity found that patients viewed such a program positively [21]. Identification of vulnerably housed patients in particular could help connect patients to rental assistance and eviction protection programs to maintain housing and prevent frank homelessness [22]. Indeed, the importance of screening for social determinants of health, including housing insecurity, has been recognized by national organizations, and healthcare systems are now subject to regulations by the Centers for Medicare and Medicaid to promote such screenings [23]. Social determinants of health screening can be embedded in the electronic medical records to enhance sustainability [24,25].

While many participants in our study were comfortable discussing their housing status with medical providers, those who were not expressed fear of stigmatization. Training in trauma-informed care is an opportunity for healthcare professionals who may be conducting housing and other social determinants of health screenings to mitigate the experience of stigma [26]. Alternatively, utilizing peer navigators or community health workers for housing assessments may offer a patient-centered solution [27]. At a minimum, providers should recognize the value of assessing housing status in all inpatients and explore this and other social determinants of health in a nonjudgmental and empathetic manner. Cultivating an awareness of, and empathy for, the extreme hardships and barriers to health among hospitalized patients living without secure housing represents an opportunity and an important step in improving care for a population that experiences profound disparities in health outcomes.

Our results demonstrate the impact of volatile interpersonal relationships in the generation and propagation of housing insecurity, a finding that is consistent with prior research [28]. Assessing patient support networks in patients with housing insecurity to identify opportunities or barriers related to social capital during hospitalization may be particularly important for effective discharge planning.

Recommendations for hospitals and healthcare systems

Our study highlights the complex needs of patients with housing insecurity. PEHs are often entangled in cycles of poor health, substance use, and hospitalization, and vulnerably housed patients are at risk of hospital-generated housing insecurity. Hospital systems should ensure adequate staffing of critical inpatient social work teams and consider investing in specialty social work teams with enhanced capabilities to navigate local housing resources [29]. Furthermore, healthcare systems should actively partner with community organizations to address local housing capacity and resources [30]. During the COVID-19 pandemic, hospital systems partnered with local hotels to provide PEHs with a place to isolate after a diagnosis of COVID-19; expanding on this model to provide patients with a safe place to recuperate after a hospital stay has the potential to benefit patients and hospital systems alike [31,32].

Substance use contributed to the need for hospital care for many patients in this study, which is consistent with prior data demonstrating that PEHs experience higher rates of hospitalization for substance use and mental health disorders than their housed counterparts [5]. Hospitalization provides a unique opportunity for providers to address substance use through inpatient addiction medicine consultation services [33-35]. However, substance use disorders may be refractory to medical treatment until stable housing is achieved as many patients utilize substances as a coping mechanism for the extraordinary hardships of homelessness. For patients who are unable to break the cycle of substance use and housing insecurity, a housing-first model can be highly efficacious [36]. Comprehensive sober living programs represent a powerful tool for some patients experiencing housing insecurity and comorbid substance use [37]. Alternatively, for patients who are unable to commit to total abstinence from substance use, hospitalization can be leveraged to provide interventions that prioritize harm reduction [38]. An investment in inpatient addiction medicine programs that integrate social work services to provide specific resources to address housing insecurity as well as substance use disorders could provide patients an ideal opportunity to achieve both stable housing and entry into recovery.

Recommendations for broader health policy

To address the vicious cycle of poor health, hospitalization, and housing insecurity described in this study, strategies to prevent entry into homelessness are key. Eviction prevention and rental assistance programs, as well as increased state and federal funding for a range of affordable and supportive housing options, can help reduce patients’ risk of entering into homelessness [22]. A concerted investment in coordination between community partners to reduce barriers to engagement with local and regional resources also holds potential to improve outcomes for patients across a range of housing-insecure situations [39]. For those already experiencing homelessness, having a safe space to recuperate after a hospital stay has the potential to break the cycle of housing insecurity and poor health. To this end, increased local and regional investment in robust medical respite care is necessary to promote recuperation post-hospitalization [40].

Strengths and limitations

This qualitative study’s strengths lie in its unique ability to provide insights into patient-centered opportunities to improve care for a group of patients with limited ability to advocate for their needs in the healthcare system. Our recruitment strategy, which involved approaching a random selection of patients without prior knowledge of housing status, ensured a breadth of perspectives and insights, including patients who otherwise may not have been identified as housing insecure. Our results demonstrated the shared and divergent experiences of both homeless and vulnerably housed patients.

One of the limitations of this study is that the participants were interviewed at variable times during their admissions, which influences their experience in the hospital. Exclusion of non-English speaking patients limits the applicability of our findings. Finally, a significant proportion of patients approached declined to participate or were unable to consent to participation. We did not delineate between being unable to participate versus declining to participate in our data collection process, which could bias our results as those with chronic cognitive impairment or delirium - conditions more common in the geriatric population - could have been selectively excluded. Furthermore, we did not collect data on the reasons that patients declined participation. We would hypothesize that those who were stably housed may have preferentially declined participation due to a lack of interest in the subject matter; notably, these patients would in any case have been excluded from a full interview after screening. Other reasons for non-participation could include the low compensation that was offered ($5) or feeling too ill to participate on the day approached. Regardless, this process of self-selection also introduces the possibility of bias in our results.

## Conclusions

This qualitative study of vulnerably housed and frankly homeless medical inpatients underscores the importance of the cyclical and multifaceted relationship between unstable housing, hospitalization, and poor health, and the complexity of factors that perpetuate this relationship. Housing insecurity may be underappreciated in hospitalized patients and is likely to increase with the economic fallout and widening healthcare disparities exacerbated by the COVID-19 pandemic. Given the acute healthcare utilization patterns of patients experiencing housing insecurity, hospital systems and hospital-based healthcare providers are well-positioned to advocate for, and provide, tailored patient-centered care to this population. Additionally, hospitals and health systems have an opportunity to improve health equity through strategic investments in resources to address housing insecurity in hospitalized patients.

## Figures and Tables

**Figure 3:**
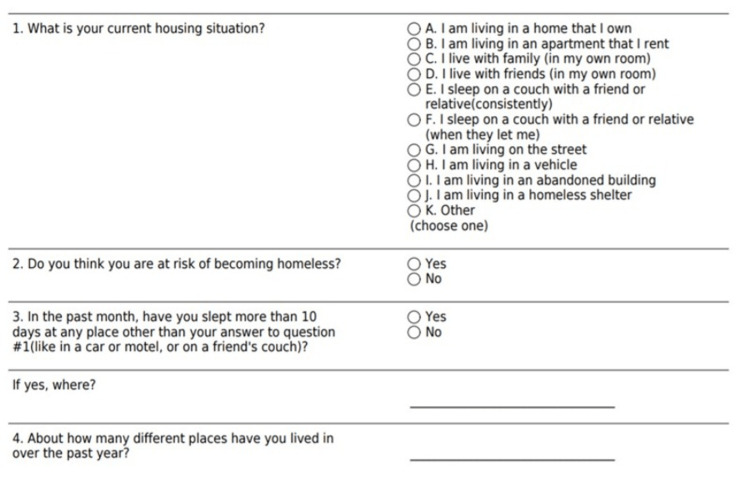
Screening survey for housing insecurity

**Figure 4:**
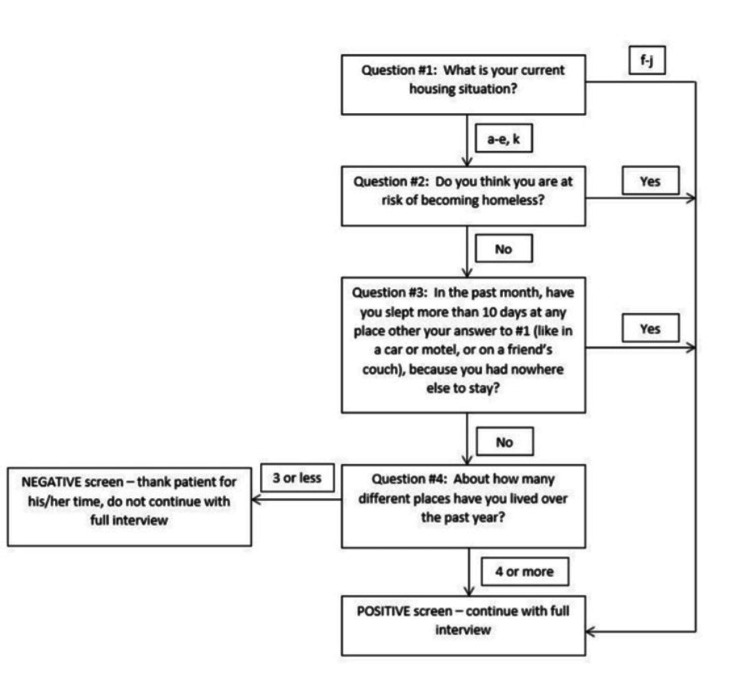
Interpretation of screening

## Appendices

Appendix 1:  Screening survey for housing insecurity

Appendix 2:  Interview guide

**Table 4:** Interview guide

Open-ended questions:
1. Tell me more about your housing situation.
2. Do you have any concerns about your housing (safety, affordability, stability)?
a. If yes, what concerns do you have?
3. Can you tell me more about how you feel your current housing situation affects your health, and any challenges you face because of it?
4. What is your biggest concern about being cared for in the hospital?
5. What is your biggest concern when you leave the hospital?
6. Do you have any suggestions for improving the care that you received in the hospital or after you leave the hospital?
7. Do you feel that you have been cared for differently because of your current housing situation on this hospitalization?
8. Have your doctors or other members of your medical team asked you about your current housing situation during this hospitalization? If yes, who and what did they ask you? If no, why do you think they didn’t ask?
9. What things do you wish your hospital doctors knew about your housing situation or other challenges you face which you feel could help them take better care of you?
10. What would make you more comfortable discussing housing status with your medical providers?
11. What resources (such as lists of shelters, community centers, etc.) could we offer in the hospital that you think would be helpful?
Discrete Questions:
1. How many people do you currently live with, not including yourself?
2. How much of your income do you spend on housing?
3. Over the past year, have you had problems with any of the following at the place where you live:
a. No heat or not enough heat to live comfortably
b. No electricity or not enough electricity to live comfortably
c. No running water or not enough water to live comfortably
d. No toilet or a non-functioning toilet
4. How much do you believe your current housing situation affects your health?
5. How comfortable do you feel discussing issues related to your housing situation with your hospital care team?
6. Has anyone during this hospitalization offered you resources such as a list of shelters, community centers, and food banks or other resources?
a. If yes - were these resources helpful?
7. How do you get to medical appointments? (e.g., walk, car, bus, bicycle)
8. In the past year, have you missed any medical appointments due to a lack of transportation?
9. Are you currently working?
10. How many jobs have you worked in the past 12 months?
11. In the past year, have you ever run out of food?
12. In the past year, have you ever been unable to fill a prescription because you did not have enough money to fill the prescription?
13. In the past year, have any of your medications been lost or stolen?
14. In the past year, have you had to miss or cancel a medical appointment because you were unable to pay for it?
15. Is there someone you can rely on to help you if you have medical problems or other concerns?
16. Do you have a case manager or another designated person who is not a family member or friend who can help you with problems? If yes, who
